# Precision evaluation of 2 CT-based radiostereometric analysis systems in a cadaver study

**DOI:** 10.2340/17453674.2025.44949

**Published:** 2025-11-25

**Authors:** Maaike R DE BONDT, Frank-David ØHRN, Lars H W ENGSETH, Anselm SCHULZ, Bart L KAPTEIN, Stephan M RÖHRL, Petra J C HEESTERBEEK

**Affiliations:** 1 Research Department, Sint Maartenskliniek, Nijmegen; 2 Department of Biomechanical Engineering, University of Twente, Enschede, The Netherlands; 3 Orthopaedic Department, Nordmøre and Romsdal Hospital, Møre and Romsdal Hospital Trust, Kristiansund; 4 Faculty of Medicine and Health Sciences, Department of Neuromedicine and Movement Science (INB), NTNU Norwegian University of Science and Technology, Trondheim; 5 Division of Orthopaedic Surgery, Oslo University Hospital Ullevål (CIRRO), Oslo; 6 Faculty of Medicine, University of Oslo, Oslo; 7 Department of Radiology and Nuclear Medicine, Oslo University Hospital Ullevål, Oslo, Norway; 8 Department of Orthopaedics, Leiden University Medical Center, Leiden, The Netherlands

## Abstract

**Background and purpose:**

To address the limitations of radiostereometric imaging and to eliminate the need for intraoperative marker placement, CT-based radiostereometric analysis (CT-RSA) software systems have been developed. We aimed to evaluate the precision of a novel CT-RSA software system, V3MA, against an established CT-RSA software system, CTMA, while also examining the impact of CT scanner model on precision.

**Methods:**

7 CT scans per scanner (Siemens SOMATOM Force and GE Revolution) of a porcine cadaver with a knee implant were used for pairwise comparisons. By aligning paired CT scans, the translation (mm), rotation (°), and maximum total point motion (MTPM, mm) of the tibial implant with respect to the bone were computed. V3MA aligned the scans using the voxel gray values of the bone and implant, whereas CTMA aligned the surface points of the bone and implant. The precision of both software systems and the effect of both scanner models were investigated using paired data in a linear mixed model.

**Results:**

Both software systems resulted in a similar MTPM (contrast V3MA–CTMA –0.002 mm, 95% confidence interval [CI] –0.015 to 0.011; V3MA: 0.09 mm; CTMA: 0.10 mm), indicating comparable precision using a minimal important difference of 0.10 mm. Using Siemens scanner data resulted in a higher estimated MTPM than using GE scanner data (contrast Siemens–GE 0.046 mm, CI 0.024–0.067; Siemens: 0.12 mm; GE: 0.072 mm).

**Conclusion:**

The precision of the new CT-RSA software system, V3MA, is comparable to that of CTMA under zero-motion assumptions. Minor, clinically irrelevant, inter-scanner differences in CT-RSA precision exist for both software systems.

Aseptic loosening is one of the most frequent reasons for total knee arthroplasty (TKA) revision, accounting for approximately 15–30% of all knee revisions [1,2]. Radiostereometric analysis (RSA), with a precision of 0.05–0.5 mm, is an effective tool for measuring implant migration, enabling the proxy assessment of (late) implant loosening [3].

Despite the high accuracy and precision of RSA, some limitations exist. RSA is not feasible for every TKA patient. Although model-based RSA (MBRSA) has eliminated the need for markers on implants, it still requires intraoperative implantation of tantalum markers in the bone [4]. This process is time-consuming and thus costly. For RSA imaging, specialized equipment and trained personnel are necessary, limiting its widespread use and adding costs [4,5]. Additionally, markers might be obscured on radiographs by a large (revision) implant, leading to inaccurate results or missing values [5-7].

To address these limitations, CT-based RSA (CT-RSA) methods have been developed. These techniques do not require intraoperative marker placement or specialized traditional RSA equipment. This offers potential clinical benefits by enabling migration and inducible displacement analysis in any hospital equipped with a CT scanner. By comparing CT scans taken at different time points, implant migration can be measured. One CT-RSA software system, CT-based Micromotion Analysis (CTMA) (Sectra, Linköping, Sweden), has demonstrated promising precision in several studies, but a CT scanner model may influence precision [5,7-11]. Another CT-RSA software system, Volumetric Matching MicroMotion Analysis (V3MA) (RSAcore, LUMC, Leiden, the Netherlands), has recently been developed [12]. We aimed to compare the precision of the 2 CT-RSA software systems (V3MA and CTMA) using MBRSA as a gold standard and to investigate whether the CT scanner model affects precision. It is hypothesized that V3MA will demonstrate precision comparable to CTMA, with potential differences in precision between CT scanners.

## Methods

### Study design

This equivalence study was reported using the Guideline for RSA and CT-RSA Implant Migration Measurements [13]. Data was re-used from a porcine knee implant cadaver study conducted by Engseth et al. that was approved by the local research committee of Oslo University Hospital [5]. This dataset included 1 porcine cadaver with a NexGen CR tibia size 4, 10 mm insert, and femur size C (Zimmer Biomet, Warsaw, IN, USA) total knee implant. This implant was captured using RSA imaging and 2 different CT scanners (GE Revolution, GE Healthcare, Chicago, USA, and Siemens SOMATOM Force, Siemens Healthcare GmbH, Erlangen, Germany).

### Porcine model and scanning procedure

The porcine cadaver was scanned in 7 different positions on each modality. As these measurements were conducted on the same day, no migration of the knee implant between scans was assumed. By comparing each exposure with the following exposures (1 vs 2, 1 vs 3, …, 1 vs 7, 2 vs 3, 2 vs 4, etc.), 21 migration analyses were performed for RSA and for each CT scanner. CT protocol settings were voxel size 0.390625 x 0.390625 x 0.625 mm, slice thickness 0.625 mm, tube settings were 120 kVp and 100 mAs. Model-based iterative reconstruction algorithms (ADMIRE 2, Siemens and ASiRV50, GE Healthcare) were used, with metal artifact reduction (MAR). The effective radiation dose was estimated at 0.08 mSv (95% confidence interval [CI] 0.076–0.082). Further details on scanning settings can be found in Engseth et al. [5]. CTMA analyses were performed using 5 feature points on the implant: tip, and medial, lateral, anterior, and posterior of the baseplate. The results from CTMA analyses and MBRSA analyses of the tibial implant as conducted by Engseth et al. were available for comparison with V3MA analyses [5].

### Data preparation

**Figure 1:**
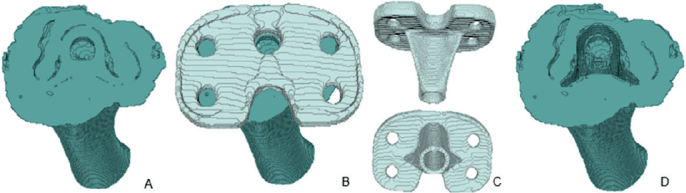
Illustration of the process of the creation of the bone and tibia implant model. A) Dilated (1 x 1 x 1 pixels) bone model, excluding tibial tuberosity and all axial slices that contain the baseplate, from superior viewpoint. B) A + dilated registered tibial implant, from superior viewpoint. C) Registered tibial implant from both posterior (upper) and inferior (lower) viewpoint. D) Dilated bone model (A) minus the registered tibial implant (C), from superior viewpoint. This is the final tibia bone model used for the analyses.

Prior to conducting V3MA analysis, the migrating object (tibial implant), the reference object (tibia bone), and the align object (tibia bone or entire knee) needed to be identified on the baseline CT scan. For analysis with V3MA, registration was performed in 2 steps: initial registration, using an align object, and final registration of the migrating and reference object. Segmentations of the tibial cortical bone and tibial implant were performed using threshold-based methods in 3D Slicer (version 5.4.0; https://www.slicer.org/), with thresholds set at 200 HU for the bone and 2,200 HU for the implant [5,14,15]. The segmentations were manually refined to exclude femur bone, femur implant, and tantalum markers. Additionally, the tibial tuberosity was not fully fused with the tibial bone and therefore may theoretically have moved between scans. Consequently, it was decided to erase this part from the tibial segmentation. All slices covering the tibial baseplate were also excluded from the bone segmentation, as scattering in this region reduced reliability of the segmentation. The resulting segments (bone and implant) were then dilated by 1 pixel (0.4 x 0.4 x 0.6 mm). The CAD model of the tibial implant was registered at the segmentation of the tibial implant and then dilated and used as migrating object. To prevent overlap, the registered tibial implant was subtracted from the bone model before the bone model was used in V3MA (Figure 1). The entire knee, used for initial alignment, was generated by thresholding at 3 HU and excluding the CT scanner bench. CT scans from both scanners were analyzed using the same data preparation steps, except for the align object. For the GE scanner, alignment was performed using the tibia bone model as the align object. However, this approach failed in visually acceptable alignment for most Siemens scanner data, necessitating the use of the entire knee as the align object in all Siemens scanner data.

### V3MA protocol

**Figure 2:**
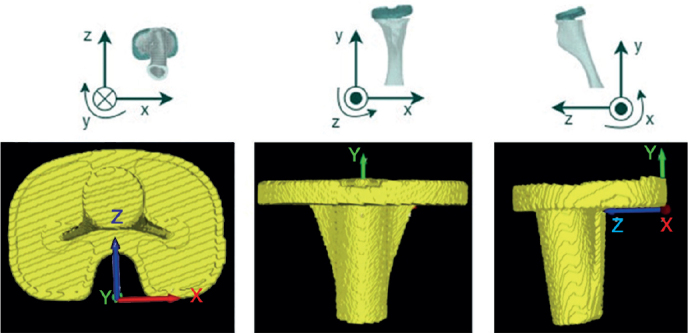
Position and orientation of the coordinate system used in V3MA as shown in the different 3D views (left: inferior, middle: anterior, right: medial). These planes are slightly tilted compared with the general MBRSA coordinate system. The general MBRSA coordinate system is shown in the upper row: x-direction: medial positive for right-sided knee; y-direction: proximal positive; and z-axis: anterior positive.

CT scans were analyzed with a recently developed CT-RSA software system, V3MA (version 0.1.0, RSAcore, Leiden, The Netherlands) [12]. For comparison of CT-scans, the following steps were performed:

1. 2 CT scans were loaded into the software, 1 as baseline and 1 as follow-up, as well as the models, corresponding to the baseline CT, of the tibial implant and tibia bone and, if necessary, the entire knee.
2. Points were marked on the baseline image to create a coordinate system or serve as feature points for migration calculation (see Supplementary data, Figure A).
3. Scans were fully automatically aligned and matched, resulting in calculation of the translation, rotation, and maximum total point motion (MTPM) of the tibial implant between the scans. Translations (mm) and rotations (°) were given along the x- (medial), y- (superior), and z-axis (anterior) (Figure 2), and as a combined total translation (TT) or total rotation (TR). TT and TR were calculated using the 3D Pythagorean theorem.

### Coordinate system

In MBRSA analysis, the model coordinate system of the tibia was used. Similarly, in CTMA analysis, a comparable coordinate system was applied. However, its origin differed. To enable comparison of V3MA with both MBRSA and CTMA, the coordinate system of V3MA was defined in a similar manner, aligning its origin with that of CTMA (see Figure 2).

### Outcome measures

The primary outcome parameter in this comparison study is the precision of the measured MTPM in mm (i.e., “the length of the translation vector corresponding to the point on the prosthesis that has moved most, disregarding its location” [13]) using V3MA. This precision is compared with the maximum translation of the 5 selected feature points measured using CTMA and the MTPM as measured using MBRSA. It is widely accepted, and shown by Engseth et al. 2025, that this maximum translation of the 5 selected feature points is another method to calculate MTPM and is therefore called MTPM throughout the remainder of this paper [11,13,16]. Note that these values were called maximum total translation (max TT) in the 2023 paper by Engseth et al. [5]. The secondary outcome measure was the comparison of the precision of 2 different CT scanner models, also expressed in MTPM (mm).

### Statistics

All statistical analyses were conducted using R (version 4.4.0, R Foundation for Statistical Computing, Vienna, Austria) and RStudio (version 2024.4.1.748) using packages lme4, dplyr, ggpubr, emmeans, and ggplot2 for data analysis and visualization.

A linear mixed-model analysis was performed using paired data to evaluate the difference in precision between the V3MA and CTMA software systems and both scanner models. Software and scanner were selected as fixed effects, CT scan pair ID as random effect. As the interaction between software and scanner did not contribute significantly, it was not included in the final model. Model estimates (least squares means) and differences (contrasts) were presented with 95% confidence interval [CI] (±1.96*SD) [17].

**Figure 3:**
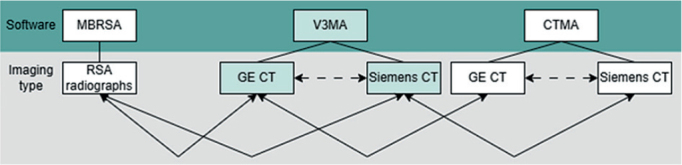
Comparisons made between the different software systems and imaging modalities. Solid line arrows represent the primary analyses; dashed line arrows represent the secondary analyses.

Additionally, the experimental MTPM values were compared. To evaluate inter-software differences, the 21 outcomes (MTPMs) were compared pairwise, per scanner. This paired comparison was made to evaluate both systematic errors (mean difference) and random errors (standard deviation of differences). Similarly, pairwise comparisons were made between V3MA and the MBRSA measurements and between the 2 scanner models (Figure 3).

To verify whether the MTPMs of V3MA, CTMA, and MBRSA followed a normal distribution, the Shapiro–Wilk test was performed. Paired t-tests or nonparametric Wilcoxon signed-rank tests were used, where appropriate, for comparisons of experimental values. CIs of comparisons between scanners or software systems were reported as mean ±1.96*SD. Comparisons with CTMA and MBRSA were performed using the results of Engseth et al. [5], and are specifically indicated. Although RSA guidelines recommend using the SD as the measure of precision, various precision metrics have been reported in the literature [13]. To facilitate comparison, in the present study the used measure per study was provided in brackets.

An increase in MTPM at group level exceeding the threshold of 0.2 mm between the first and second year post-surgery may predict early loosening of the implant [18]. Based on this, Øhrn et al. proposed that an MTPM of 0.^10^ mm should be the upper limit of an acceptable clinical difference in precision between 2 methods for measuring migration in TKA [19]. We therefore state the minimal important difference (MID) in MTPM as 0.10 mm. When the CI of the MTPM difference does not include this ±0.10 mm margin, comparability of precision may be concluded [20].

### Data sharing, funding, use of AI tools, and disclosures

The dataset “V3MA migration analysis” is available through: 10.5281/zenodo.17522095. AI-based tools were used for English language editing and improving the clarity of some sentences only. Apart from that, no AI was used. The authors have not received any funding and declare no conflicts of interest. Complete disclosure of interest forms according to ICMJE are available on the article page, doi: 10.2340/17453674.2025.44949

## Results

**Figure 4:**
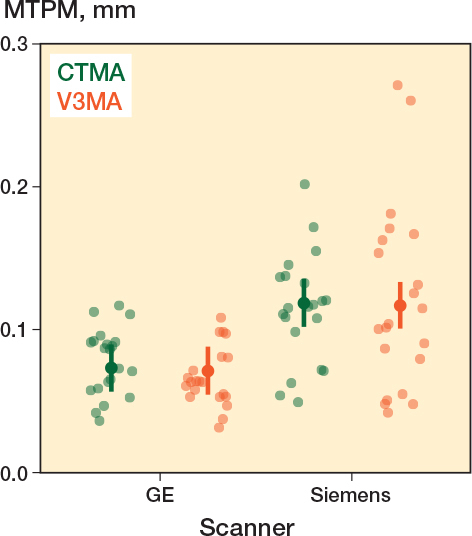
Predicted mean values (estimates) by the linear mixed model. Vertical lines indicate the 95% confidence interval of the predictions. Separate data points (dots) represent the source data on which the model was built.

Analysis with V3MA resulted in a 0.002 mm (CI –0.015 to 0.0^11^) smaller estimated MTPM compared with the CTMA analysis. Thus, both software systems resulted in a similar MTPM estimation (V3MA: 0.094 mm, CI 0.082–0.11; CTMA: 0.096 mm, CI 0.083–0.11), indicating similar precision. When data from the Siemens scanner was used, the estimated MTPM increased by 0.046 mm (CI 0.024–0.067) compared with GE scanner data (Siemens: 0.12 mm, CI 0.10–0.13; GE: 0.072 mm, CI 0.057–0.088). The marginal R^2^ of the model was 0.239 and the conditional R^2^ was 0.586, indicating that random effects contributed substantially to the variance explained. MTPM predictions of the model are shown in Figure 4.

### Inter-scanner differences

**Figure 5:**
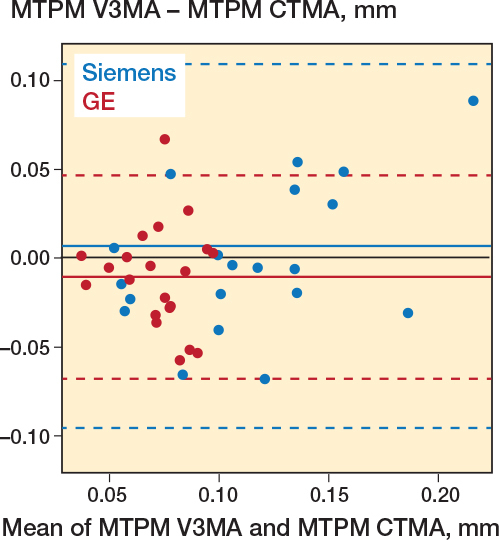
Bland–Altman plot, visualizing the differences between V3MA and CTMA, per scanner model. GE scanner outcomes are in deep-pink dots, Siemens scanner outcomes are in blue dots. Solid lines indicate the mean difference per scanner model. Dashed lines indicate the 95% limits of agreement.

A visual difference in quality between the 2 scanner models was observed during the preparation of the migrating, reference, and align objects: metal artifacts were more pronounced on the scans from the Siemens scanner than on the GE scanner scans. This difference was confirmed by comparing the precision of MTPM measured by V3MA of both scanners, resulting in a better precision for the GE scanner (difference: –0.046 mm, CI –0.067 to –0.024; Table). Figure 5 visualizes these smaller MTPM values of the GE scanner analysis compared with the Siemens scanner.

**Table:** MTPM (in mm) model estimates of the linear mixed model. Estimate (CI), including MTPM comparisons between software systems and between scanner models. Smaller MTPM values indicate better precision

Item	GE Estimate (CI)	Siemens Estimate (CI)	Contrast (CI) GE–Siemens
V3MA	0.071 (0.055 to 0.088)	0.12 (0.10 to 0.13)	–0.046 (–0.067 to –0.024)
CTMA **^a^**	0.073 (0.057 to 0.090)	0.12 (0.10 to 0.14)	–0.046 (–0.067 to –0.024)
Contrast (CI)			
V3MA–CTMA	–0.002 (–0.015 to 0.011)	–0.002 (–0.015 to 0.011)	

CI: 95% confidence interval.

a As published previously by Engseth et al.

### Experimental data

Higher precision for MTPM (expressed by lower values) was measured with V3MA on the GE scanner compared with MBRSA (0.58 mm, CI 0.20–0.96; difference: –0.51 mm, CI –0.60 to –0.42), see Supplementary data (Table A). More detailed results of the experimentally measured migration values, categorized by software, scanner, coordinate system direction, and individual feature points, are shown in the Supplementary data (Tables A–C).  

## Discussion

We aimed to evaluate the precision of a novel CT-RSA software system, V3MA, against an established CT-RSA software system, CTMA, while also examining the impact of the CT scanner model on precision. We found that the precision of MTPM measurements obtained using V3MA is comparable to the precision of MTPM measurements derived from CTMA and superior to that of MBRSA. Furthermore, CT scans conducted with a GE scanner showed better precision than those performed with a Siemens scanner when analyzed using V3MA or CTMA.

In the context of the MID of 0.10 mm, the comparison of V3MA precision with the CTMA analysis in this study (difference 95% CI lower limit GE: –0.015 mm; Siemens: –0.015 mm) falls well within this threshold, suggesting its feasibility in clinical (research) practice. Additionally, the statistically significant inter-scanner difference, with a 95% limit of 0.067 mm, represents an acceptable clinical variation between these scanners. As such, this difference is not considered clinically relevant.

Our results (±1.96*SD) are not only comparable to the precision findings of Engseth et al. (±1.96*SD) but also to the V3MA precision in TKA reported by de Laat et al. (SD), which ranged between 0.01 and 0.06 mm for translations and between 0.02° and 0.07° for rotations [12]. However, these values are SDs from individual translations and rotations, and the SD of MTPM was not presented by de Laat et al.

It is important to consider that the 2 CT-RSA methods utilize different points on the tibial implant to calculate migration. V3MA, which uses all implant points, was expected to result in higher MTPM values than the version of CTMA used in the study by Engseth et al., which relied on 5 selected feature points [5]. However, the findings did not support this assumption, as both methods produced comparable MTPM values (see Table). It should be noted that in the currently available version of the CTMA software, MTPM is easily accessible based on all implant points. Additionally, V3MA allowed for an alternative calculation of MTPM using selected feature points, which demonstrated minimal differences when compared with MTPM derived from all implant points. Specifically, for GE scanner data, the discrepancy was only 0.01 mm (CI 0.00–0.02), whereas Siemens scanner data showed a slightly greater difference, yet still clinically insignificant. The slightly higher mean MTPM values for Siemens scanner data may explain this minor discrepancy. These results are in line with the findings of Engseth et al. (2025), as their study using the upgraded CTMA software found no statistically significant difference between maximum TT and MTPM within the first year [11].

Another notable observation was the influence of metal artifacts, which were more pronounced in Siemens CT scans than in GE scans. Analysts of both CTMA and V3MA software observed these artifacts, and analyses of Siemens scans were found to be less precise regardless of the software used. It is hypothesized that these metal artifacts negatively impact migration precision by reducing the quality of alignment during analysis. Although the inter-scanner difference was not clinically relevant, for research purposes attention is needed when interpreting migration results of scans obtained from different CT scanner models. When follow-up scans are acquired on a different scanner, especially one introducing greater metal artifacts, the resulting increase in apparent migration may reflect artifact-induced variability of migration measurements rather than true implant migration.

Proper initial alignment is important for image registration. In CTMA, surface points are indicated in both the baseline as well as the follow-up CT scan. This allows for easy initial alignment. In V3MA, feature points can also be used for alignment, or, as an alternative, a segmented model of only the baseline CT scan is used. In our study, the segmented model was used, making initial alignment more difficult. Additionally, the use of different alignment models (tibial bone or entire knee) to optimize computational speed, especially when CT scan quality is relatively low or when large rotational variations between CT scans are present, might have influenced the outcomes. However, in Siemens scanner cases where the tibial bone model was sufficient for proper alignment, the migration outcomes were consistent with those obtained from the same cases using the entire knee for alignment. This suggests that migration outcomes are more dependent on qualitative alignment rather than the specific alignment approach that was used. These findings highlight the necessity of visually verifying alignment before performing migration calculations to obtain reliable migration data.

Furthermore, no established quality control measure for CT-RSA analysis has been published to date. This measure should have a similar role to that which the condition number (CN) and mean error of rigid body fitting (ME) have in MBRSA. Consequently, developers and researchers might also focus on developing a transparent and objective CT-RSA quality control method.

### Clinical perspective

The finding that both CT-RSA software systems (V3MA and CTMA) demonstrate better precision than model-based RSA suggests promising potential for its application in clinical research. However, superior precision alone does not necessarily imply greater accuracy, as systematically lower outcome values could mask implant migration. The data of the present study cannot confirm the presence of systematic errors. While De Laat et al. did not find any relevant systematic errors in vitro, future studies should prioritize evaluating the clinical sensitivity of V3MA in detecting migration [12].

In contrast to MBRSA, CT-RSA does not require the implantation of intraoperative markers, enabling retrospective analyses if an early postoperative CT scan exists. Although higher radiation exposure compared with conventional RSA imaging may limit clinical use, low-dose CT-RSA studies have demonstrated encouraging outcomes [8,16,19]. Furthermore, the availability of multiple CT-RSA software options can stimulate innovation, expand user choice, improve quality and reliability, and might reduce costs.

### Strengths

A major strength of the current study is that it directly compares the precision of 2 different CT-RSA software systems on the same dataset. This approach eliminates potential discrepancies stemming from variations in experimental execution.

### Limitations

First, as different teams have performed the analyses and no cross-validation by a single analyst occurred, potential analyst-related variability cannot be excluded. Nevertheless, no statistical differences were found despite having different analysts. Therefore, we expect the analyst-related variability to be negligible.

Second, the positioning of the porcine cadaver was not standardized and could have varied slightly between the 2 scanners. As a result, inter-scanner analyses were conducted differently than inter-software comparisons. For inter-software analyses, identical CT scans were available, allowing examination of software differences using the exact same comparisons. In contrast, for inter-scanner analyses, only group outcomes could be compared, as individual pose variations prevented direct comparisons.

Third, the use of a porcine cadaver rather than a human cadaver may have influenced the results. While the size of a porcine knee closely approximates that of a human, its morphology differs. The asymmetry of the porcine tibia could impact the accuracy of CT scan alignment, potentially leading to less precise outcomes when applied to human cadavers, as the human tibia exhibits greater symmetry.

Finally, the 21 comparisons (per scanner) used were derived from a single cadaver and only 7 CT scans, meaning that they are not independent. This is a possible weakness of the study, as it does not include 15 fully independent cases [17]. Using only 1 porcine cadaver limits the generalizability of this study due to the lack of anatomical variation. However, the study aimed to utilize existing data optimally without creating new datasets, and a porcine cadaver is deemed superior to an artificial phantom.

### Conclusion

The precision of a new CT-RSA software system, V3MA, is comparable to that of CTMA under zero-motion assumptions. Minor, clinically irrelevant, inter-scanner differences exist regarding the precision of the V3MA software.

*In perspective*, even though the V3MA software seems promising, clinical comparisons with the current standard MBRSA should be performed prior to its implementation in clinical research practice.

### Supplementary data

Tables A–C and Figure A are available as supplementary data on the article page, doi: 10.2340/17453674.2025.44949

## Supplementary Material



## Notes

MB: conceptualization, methodology, formal analysis, investigation (V3MA), data curation, writing—original draft, writing—review and editing. FØ: formal analysis, investigation (CTMA, MBRSA), writing—review and editing. LE: investigation (CTMA, MBRSA), writing—review and editing. AS: investigation (CTMA, MBRSA), writing—review and editing. BK: conceptualization, software (V3MA), writing—review and editing. SR: conceptualization, writing—review and editing. PH: conceptualization, methodology, formal analysis, writing—review and editing, supervision.

Handling co-editors: Ivan Hvid and Robin Christensen

*Acta* thanks Hannu T Aro and Olof Sköldenberg for help with peer review of this manuscript.
