# Supplementary material for: Precision evaluation of 2 CT-based radiostereometric analysis systems in a cadaver study
Source: Acta Orthop. 2025 Nov 25;96:867–72. doi: 10.2340/17453674.2025.44949 (PMC12645134; doi:10.2340/17453674.2025.44949)
Supplement: Supplementary file 1 [file ActaO-96-44949-s1.pdf]

Supplementary Material

Table A. Outcome of experimental precision measurements for V3MA method, as mean (CI), including MTPM comparisons between software systems and between scanner models. Smaller MTPM values indicate better precision

	V3MA GE			V3MA Siemens				CTMA GE [a]	CTMA Siemens [a]		MBRSA [a]
	mean [CI]	V3MA GE minus CTMA GE [CI]	V3MA GE minus MBRSA [CI]	mean [CI]	V3MA Siemens minus CTMA Siemens [CI]	V3MA Siemens minus MBRSA [CI]	V3MA GE minus Siemens [CI]	mean [CI]	mean [CI]	CTMA GE minus Siemens [CI]	mean [CI]
**MTPM, mm**	0.07 [0.03–0.11]	–0.011 [–0.024 to 0.003]	–0.511 [–0.603 to –0.420]	0.12 [0.00–0.25]	0.007 [–0.017 to 0.030]	–0.457 [–0.555 to –0.359]	–0.054 [–0.085 to –0.023]	0.08 [0.03–0.12]	0.11 [0.04–0.19]	–0.037 [–0.053 to –0.020]	0.58 [0.20–0.96]

MBRSA: model–based radiostereometric analysis (RSA). CI: 95% confidence interval.

[a] as published previously by Engseth et al.

Table B. Values of experimental precision measurements for V3MA method per direction of translation or rotation and as TT and TR. Smaller MTPM values indicate better precision. MBRSA, model-based radiostereometric analysis (RSA)

	V3MA GE			V3MA Siemens			CTMA GE [a]	CTMA Siemens [a]	MBRSA [a]
	mean [CI]	V3MA GE minus CTMA GE [CI]	V3MA GE minus MBRSA [CI]	mean [CI]	V3MA Siemens minus CTMA Siemens [CI]	V3MA Siemens minus MBRSA [CI]	mean [CI]	mean [CI]	mean [CI]
**Translation, mm**									
**Medial**	–0.02 [–0.08 to 0.04]	–0.018 [–0.024 to –0.002]	–0.009 [–0.084 to 0.065]	0.00 [–0.06 to 0.05]	–0.007 [–0.020 to 0.006]	0.006 [–0.072 to 0.085]	0.00 [–0.06 to 0.06]	0.00 [–0.04 to 0.05]	–0.01 [–0.33 to 0.31]
**Proximal**	0.01 [–0.03 to 0.04]	0.013 [0.001 to 0.025]	–0.002 [–0.030 to 0.025]	–0.03 [–0.16 to 0.11]	–0.036 [–0.084 to 0.012]	–0.036 [–0.083 to 0.010]	0.02 [–0.06 to 0.10]	–0.03 [–0.11 to 0.06]	0.01 [–0.13 to 0.14]
**Anterior**	0.00 [–0.03 to 0.03]	–0.021 [–0.039 to –0.002]	–0.045 [–0.153 to 0.064]	–0.01 [–0.11 to 0.10]	0.019 [–0.012 to 0.049]	–0.051 [–0.148 to 0.045]	0.01 [–0.03 to 0.04]	–0.01 [–0.09 to 0.08]	0.04 [–0.41 to 0.50]
**Total translation (TT)**	0.04 [0.00 to 0.08]	–0.013 [–0.024 to –0.002]	–0.214 [–0.279 to –0.150]	0.09 [0.00 to 0.17]	0.018 [–0.002 to 0.039]	–0.169 [–0.237 to –0.101]	0.05 [0.01 to 0.09]	0.07 [0.02 to 0.11]	0.25 [–0.01 to 0.51]
**Rotation, °**									
**Transversal**	0.00 [–0.03 to 0.04]	–0.006 [–0.017 to 0.005]	–0.318 [–0.500 to –0.135]	–0.03 [–0.21 to 0.15]	0.020 [0.007 to 0.033]	–0.478 [b] [Q1: –0.661; Q3: –0.014]	0.01 [–0.03 to 0.05]	–0.05 [–0.26 to 0.16]	0.32 [–0.46 to 1.10]
**Internal**	0.01 [–0.12 to 0.13]	0.037 [b] [Q1: –0.029; Q3: 0.052]	0.132 [–0.097 to 0.361]	0.03 [–0.07 to 0.14]	0.013 [–0.014 to 0.040]	0.160 [–0.056 to 0.377]	–0.03 [–0.10 to 0.04]	0.03 [–0.10 to 0.16]	–0.13 [–1.12 to 0.87]

**Varus**	0.02<br>[–0.03 to 0.06]	0.044<br>[0.026 to 0.063]	0.009<br>[–0.134 to 0.151]	–0.03<br>[–0.10 to 0.05]	–0.059<br>[–0.097 to –0.020]	–0.034<br>[–0.189 to 0.120]	0.00<br>[–0.09 to 0.09]	–0.02<br>[–0.09 to 0.04]	0.01<br>[–0.03 to 0.64]
**Total rotation (TR)**	0.07<br>[0.01 to 0.12]	0.005<br>[–0.024 to 0.003]	–0.668<br>[–0.798 to –0.538]	0.11<br>[0.01 to 0.22]	–0.014<br>[–0.030 to 0.001]	–0.622<br>[–0.757 to –0.487]	0.06<br>[0.01 to 0.11]	0.13<br>[0.00 to 0.25]	0.73<br>[0.20 to 1.27]

CI*:* 95% confidence interval.

[a] as published previously by Engseth et al.

[b] indicates use of non–parametric test instead of t–test: median (Q1; Q3).

Table C. Values of experimental precision measurements for V3MA method for peripheral points (see supplementary materials Figure A) in mm, also per direction, including comparison between V3MA and CTMA

		**V3MA GE**		**V3MA Siemens**		**CTMA GE [a]**	**CTMA Siemens [a]**	**MBRSA [a]**
Peripheral point	**Translation direction**	**mean [CI]**	**V3MA GE minus CTMA GE [CI]**	**mean [CI]**	**V3MA Siemens minus CTMA Siemens [CI]**	**mean [CI]**	**mean [CI]**	**mean [CI]**
Tip	**Total translation**	0.03<br>[0.01 to 0.05]	–0.010<br>[–0.022 to 0.002]	0.05<br>[0.01 to 0.08]	–0.009 [b]<br>[Q1: –0.037; Q3: –0.0024]	0.04<br>[0.00 to 0.08]	0.06<br>[0.01 to 0.11]	
	**Medial**	–0.01<br>[–0.06 to 0.04]	0.001<br>[–0.006 to 0.009]	0.00<br>[–0.05 to 0.04]	0.001<br>[–0.004 to 0.006]	–0.01<br>[–0.03 to 0.02]	0.00<br>[–0.06 to 0.05]	–0.02<br>[–0.32 to 0.28]
	**Proximal**	0.00<br>[–0.03 to 0.04]	0.005<br>[–0.005 to 0.016]	–0.01<br>[–0.06 to 0.03]	0.009<br>[–0.007 to 0.024]	0.02<br>[–0.06 to 0.09]	–0.02<br>[–0.09 to 0.06]	–0.03<br>[–0.24 to 0.18]
	**Anterior**	0.00<br>[–0.02 to 0.01]	–0.021<br>[–0.038 to –0.004]	0.01<br>[–0.05 to 0.07]	0.028<br>[0.002 to 0.055]	0.00<br>[–0.02 to 0.02]	0.02<br>[–0.07 to 0.11]	–0.16<br>[–0.39 to 0.06]
Medial	**Total translation**	0.05<br>[0.02 to 0.08]	–0.015<br>[–0.026 to –0.005]	0.10<br>[–0.01 to 0.20]	–0.004<br>[–0.024 to 0.017]	0.07<br>[0.02 to 0.11]	0.10<br>[0.03 to 0.17]	
	**Medial**	–0.02<br>[–0.07 to 0.03]	–0.015<br>[–0.031 to 0.000]	0.01<br>[–0.04 to 0.06]	0.005<br>[–0.002 to 0.013]	0.00<br>[–0.07 to 0.06]	0.01<br>[–0.05 to 0.06]	–0.02<br>[–0.43 to 0.38]
	**Proximal**	0.01<br>[–0.04 to 0.06]	0.035<br>[0.014 to 0.056]	–0.03<br>[–0.14 to 0.07]	–0.061<br>[–0.114 to –0.009]	0.02<br>[–0.09 to 0.13]	–0.04<br>[–0.15 to 0.08]	–0.02<br>[–0.47 to 0.42]
	**Anterior**	0.00<br>[–0.07 to 0.06]	–0.024<br>[–0.055 to 0.006]	–0.03<br>[–0.19 to 0.14]	0.008<br>[–0.037 to 0.053]	–0.01<br>[–0.05 to 0.03]	–0.03<br>[–0.18 to 0.12]	0.11<br>[–0.63 to 0.86]
Lateral	**Total translation**	0.06<br>[0.02 to 0.09]	–0.008<br>[–0.025 to 0.009]	0.04<br>[0.01 to 0.08]	–0.014<br>[–0.025 to –0.003]	0.06<br>[0.01 to 0.12]	0.06<br>[0.02 to 0.10]	
	**Medial**	–0.02<br>[–0.07 to 0.03]	–0.013<br>[–0.028 to 0.002]	0.01<br>[–0.04 to 0.06]	0.003<br>[–0.005 to 0.011]	0.00<br>[–0.07 to 0.07]	0.01<br>[–0.05 to 0.06]	–0.02<br>[–0.37 to 0.33]
	**Proximal**	–0.01<br>[–0.05 to 0.04]	–0.014<br>[–0.025 to –0.003]	0.00<br>[–0.04 to 0.04]	0.007<br>[–0.018 to 0.031]	0.02<br>[–0.09 to 0.13]	–0.01<br>[–0.08 to 0.05]	–0.01<br>[–0.40 to 0.39]
	**Anterior**	0.00<br>[–0.09 to 0.09]	–0.016<br>[–0.044 to 0.011]	0.01<br>[–0.05 to 0.07]	0.024<br>[0.002 to 0.045]	–0.01<br>[–0.05 to 0.03]	0.01<br>[–0.08 to 0.09]	–0.02<br>[–0.74 to 0.69]

Anterior	**Total translation**	0.04 [0.01 to 0.08]	−0.008 [−0.020 to 0.004]	0.07 [0.00 to 0.14]	−0.004 [−0.017 to 0.009]	0.05 [0.02 to 0.09]	0.07 [0.01 to 0.13]	
	**Medial**	−0.01 [−0.09 to 0.06]	−0.002 [−0.019 to 0.014]	0.02 [−0.05 to 0.10]	0.008 [−0.002 to 0.017]	−0.01 [−0.08 to 0.05]	0.02 [−0.06 to 0.10]	−0.05 [−0.67 to 0.56]
	**Proximal**	0.00 [−0.04 to 0.04]	0.01 [−0.002 to 0.023]	0.00 [−0.04 to 0.03]	−0.016 [−0.035 to 0.003]	0.02 [−0.05 to 0.09]	−0.01 [−0.09 to 0.08]	−0.10 [−0.47 to 0.26]
	**Anterior**	0.00 [−0.03 to 0.03]	−0.017 [−0.034 to −0.000]	−0.01 [−0.11 to 0.10]	−0.001 [−0.041 to 0.039]	0.01 [−0.03 to 0.05]	−0.01 [−0.11 to 0.09]	0.04 [−0.41 to 0.50]
Posterior	**Total translation**	0.04 [0.00 to 0.08]	−0.024 [−0.039 to −0.009]	0.09 [0.00 to 0.17]	−0.015 [−0.034 to 0.004]	0.06 [0.01 to 0.12]	0.10 [0.03 to 0.18]	
	**Medial**	−0.02 [−0.08 to 0.04]	−0.03 [−0.051 to −0.010]	0.00 [−0.06 to 0.05]	0.005 [−0.009 to 0.019]	0.01 [−0.07 to 0.09]	−0.01 [−0.07 to 0.06]	0.05 [−0.27 to 0.37]
	**Proximal**	0.01 [−0.03 to 0.04]	0.014 [0.001 to 0.026]	−0.03 [−0.17 to 0.11]	−0.04 [−0.091 to 0.012]	0.02 [−0.07 to 0.12]	−0.05 [−0.20 to 0.11]	0.15 [−0.14 to 0.44]
	**Anterior**	0.00 [−0.03 to 0.03]	−0.025 [−0.046 to −0.004]	−0.01 [−0.11 to 0.10]	0.039 [0.006 to 0.072]	0.01 [−0.03 to 0.05]	−0.01 [−0.11 to 0.09]	0.05 [−0.41 to 0.51]

[a] as published previously by Engseth et al.

[b] indicates use of non–parametric test instead of t–test: median (Q1; Q3).

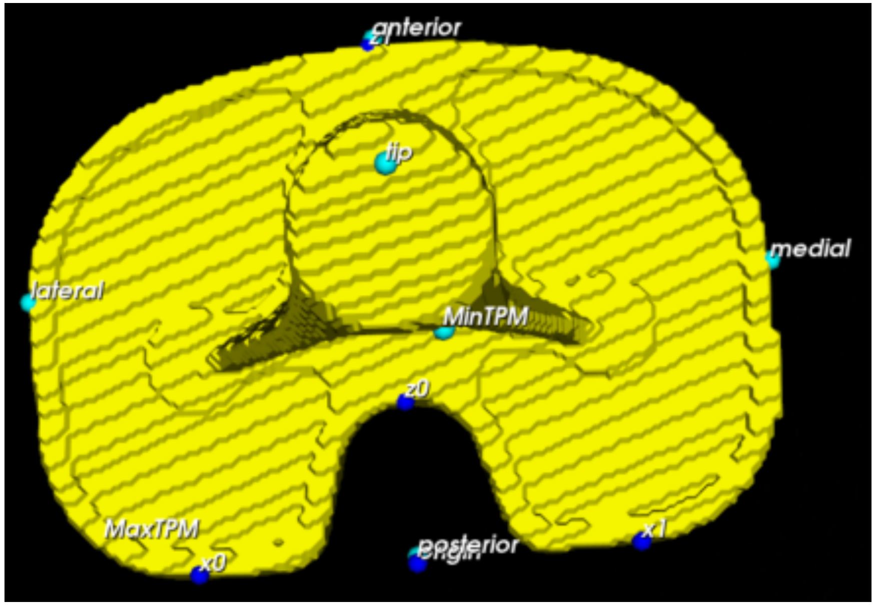

Figure A. Inferior view of all created feature points, both for establishing the coordinate system (origin, x0, x1, z0, z1) and for migration analysis (tip, medial, lateral, anterior, posterior). The z1 and anterior points are approximately in the same location, and the origin and posterior points coincide as well.
